# Temporal changes in epidemiological profile and fetal indications for late termination of pregnancy: a retrospective single-center study

**DOI:** 10.1007/s00404-021-06042-6

**Published:** 2021-04-02

**Authors:** Dana Anaïs Muin, Patricia Otte, Anke Scharrer, Gregor Kasprian, Peter W. Husslein, Herbert Kiss, Dieter Bettelheim

**Affiliations:** 1 Division of Fetomaternal Medicine, Department of Obstetrics and Gynecology, Medical University of Vienna, Waehringer Guertel 18-20, 1090 Vienna, Austria; 2 Clinical Institute of Pathology, Medical University of Vienna, Vienna, Austria; 3 Department of Biomedical Imaging and Image-Guided Therapy, Medical University of Vienna, Vienna, Austria

**Keywords:** Termination of pregnancy, Feticide, Fetal death, Congenital malformation, Epidemiology

## Abstract

**Purpose:**

To explore whether epidemiological shifts regarding reproduction and pregnancy have influenced the spectrum of indications for late termination of singleton pregnancies (TOP) above 17 weeks of gestation and to evaluate temporal changes in maternal demographics and fetal indications over the last 16 years.

**Methods:**

Retrospective single-center cohort study involving all late TOPs preceded by feticide between 1 January 2004 and 31 December 2019 at a tertiary referral hospital in Austria. Outcome variables were retrieved and a time trend assessed between two 8-year intervals (2004–2011 versus 2012–2019).

**Results:**

Between January 2004 and December 2019, a total of 209 singleton pregnancies (50.7% male; 46.9% female fetuses, 2.4% no disclosed sex) were terminated medically at a median gestational age of 25^+1^ (17^+3^–37^+1^) weeks at our institution. Predominant conditions legally justifying the late medical abortion were abnormaltities of the brain/central nervous system (*n* = 83; 39.7%), chromosomal aberrations (*n* = 33; 15.8%), complex malformations (*n* = 31; 4.8%) and abnormaltities of the musculosceletal system including diaphragmatic hernias (*n* = 18; 8.6%), as reflected by the *ICD-10-*categories “C*ongenital malformation of the central nervous system”*, “*Other congenital malformations*” and “*Chromosomal abnormalities*”. No changes were observed with regards to maternal age (30.1 ± 5.9 vs. 31.0 ± 6.0 years;  *p* = 0.315) nor frequency of assisted reproductive technologies (7.0% vs. 8.5%; *p* = 0.550). Despite a 2.5-fold increase in incidence of late TOPs, no epidemiological changes in maternal or fetal characteristics were observed over the last 16 years.

**Conclusion:**

Population profile and indications for late TOPs followed by feticide remain unchanged over time.

## Introduction

Termination of pregnancy (TOP) in the second and third trimester continuously raises ethical and moral debate on justification as to indication and methodology [1]. As per Austrian law, terminations of pregnancy above gestational week 15^+6^ are permitted upon the receipt of an institutional ethical approval for cases severely affected by maternal compromise or fetal congenital malformations considered untreatable and which may cause severe disabilities and compromise after birth [2]. The potential fetal viability around gestational week 20 often requires the iatrogenic induction of fetal cardiac arrest by ultrasound-guided injection of potassium chloride or lidocain into the cardiac chambers or umbilical vein (*feticide*) [3]. The spectrum of indications varies across Europe and so do the regulations on abortion laws, depending on national historical, political and cultural backgrounds [4]. Until recently, Ireland and Malta were the only European countries to prohibit TOP under any circumstances [5, 6]. In 2020, the Polish government has put new restrictions on the abortion law, allowing medical terminations only in cases of rape or incest and severe maternal compromise, irrespective of fetal wellbeing [7]. In the rest of Europe, induced abortions are allowed for indications related to physical and/or mental and/or socioeconomic health, mainly between 10 and 18 gestational weeks *post menstruationem* with varying incidence. The highest prevalence for late terminations is held by France with 0.97 TOPs per 1000 live births between 24^+0^ and 25^+0^ gestational weeks and 1.68 TOPs ≥ 26^+0^ gestational weeks [8].

Global demographic changes in childbearing have resulted in advanced maternal age at first childbirth, as well as higher numbers of pregnancies resulting from conception by assisted reproductive technologies (ART) [9–12]. ART has been previously linked with an increased risk of birth defects, potentially leading to late TOPs, however, recent evidence confirmed, that the risk of such was not significant after adjustment for parental risk factors, such as maternal age and co-morbidities [13, 14]. It was, therefore, our intention to explore demographic changes in our cohort of women whose pregnancies were terminated by feticide due to severe congenital malformations. Furthermore, we aimed to evaluate the spectrum of fetal malformations that resulted in late TOPs at our institution over the last 16 years.

## Methods

### Study design and data collection

We retrospectively reviewed all cases of elective late terminations of singleton pregnancies following feticide performed at the Department of Obstetrics and Fetomaternal Medicine at the Medical University of Vienna, Austria, between January 2004 and December 2019. We retrieved all maternal and fetal data as well as prenatal ultrasound reports from the electronic medical database ViewPoint Version 5.6.28.56 (General Electric Company, Solingen, Germany). Post-mortem fetal autopsy reports were derived from the hospital data system SAP GUI for Windows Version 7500.2.6.3379 (SAP NetWeaver, Austria). Prenatal ultrasound and post-mortem autopsy reports were checked for congruency and the final diagnosis supporting the late TOP was determined and classified by the “International Statistical Classification of Diseases and Related Health Problems” ICD-10-GM Version 2020 (last updated 25 May 2020) into chapters, code ranges (blocks), categories and subcategories [15].

For comparison, ICD-10-GM codes were grouped into the relevant organ systems affected by the malformation or conditions justifying the late TOP in each individual case. Organ systems were classified into brain/central nervous system (CNS), heart/circulatory system (excluding genetically confirmed DiGeorge syndrome), musculoskeletal system (including diaphragm as per ICD-10), genito-urinary system, chromosomal abnormalities, genetically confirmed microdeletion syndrome DiGeorge, metabolic (including cystic fibrosis as per ICD-10), congenital infection, detrimental amniotic fluid abnormalities, “others” (as per ICD-10) and complex malformations (in case of multi-organ involvement, such as brain and/or heart and/or further organ systems, affected by congenital malformations independent of each other).

The study period of 16 years was divided into two 8-year time intervals (1 January 2004 to 31 December 2011; 1 January 2012 to 31 December 2019). Ethnicity was self-reported by the pregnant woman at time of first visit. Maternal age was defined as age in years at the time of stillbirth. Body mass index (BMI) at first visit was grouped as underweight (≤ 18.5 kg/m^2^), normal weight (18.5–24.8 kg/m^2^), preobesity (25–29.9 kg/m^2^), Obesity Class I (30–34.9 kg/m^2^), Obesity Class II (35–39.9 kg/m^2^) and Obesity Class III (≥ 40 kg/m^2^). Smoking was defined as current smoker or non-smoker at the time of antenatal booking.

At our tertiary referral center, following initial clinical suspicion of a fetal malformation, each case undergoes a series of close follow-up visits including a fetal Magnetic Resonance Imaging (MRI), invasive genetic analysis by chorionic villous biopsy and/or amniocentesis (karyotyping, microarray, clinical exome sequencing) and further serial ultrasound scans depending on parents’ agreement, severity of the lesion and gestational week. Upon review of the multidisciplinary diagnostic reports, the parents will then receive an interdisciplinary consultation by the pediatric specialists under psychological guidance to make a fully informed consent regarding subsequent steps. If the parents decide to terminate the pregnancy after thorough consideration, their individual case will be reviewed by our institutional ethical board and judged upon from a medical and bio-psycho-social aspect as to whether to share the parents’ decision to terminate the pregnancy or not.

Following feticide and delivery, by Austrian law, each fetus must undergo conventional autopsy. At our institution, all fetal autopsies are conducted by an expert team of perinatal pathologists according to standardized guidelines at the Clinical Institute for Pathology, Medical University of Vienna, Austria. Congenital malformations are routinely photographed and documented. Congruency between prenatal suspected diagnosis of fetal congenital malformation and final postnatal autopsy and histology report is evaluated every fortnight at a local perinatology board meeting involving fetal medicine specialists, pediatricians, radiologists, geneticists and perinatal pathologists. After latest two months following stillbirth, the final diagnosis will be delivered to the parents within the frame of a post-mortem consultation. Also, potential risk factors determining the empirical recurrence risk in future pregnancies will be evaluated and discussed.

### Statistical analysis

Distribution of data was analyzed using the Kolmogorov–Smirnov test. Normally distributed variables are expressed as mean and standard deviation (M ± SD). Not normally distributed variables are expressed as median and minimum/maximum. Categorical data are given as counts (*n*) and percentages (%). Continuous data were compared with unpaired t test and Mann–Whitney *U* test, respectively. Categorical data were compared with Chi^2^ and Fisher’s Exact test, respectively, with a 99% Confidence Interval (CI). All reported *p* values are two-sided, and level of significance was set at < 0.05. Statistical tests and figures were performed with SPSS Statistics Version 26.0.0.0 (IBM Corporation, Armonk, NY, USA).

Parents’ written informed consent was obtained in each case at initial and follow-up visits as well as prior to the post-mortem examinations. The study complied with the principles outlined in the Helsinki Declaration of 1975, as revised in 2013, and was approved by the institutional review board of the Ethics Committee at the Medical University of Vienna (Registration number 1855/2017).

## Results

### Maternal and fetal baseline characteristics

Between January 2004 and December 2019, a total of 209 singleton pregnancies were terminated late with prior need of iatrogenically induced cardiac arrest at our institution. Baseline maternal and fetal characteristics are shown in Table 1. Mean (± SD) maternal age of the total study cohort at time of delivery was 30.7 ± 6.0 years. 133 (63.6%) women were of Central European ethnicity, 44 (21.1%) were Eastern European, 10 (4.8%) were Turkish, 6 (2.9%) were from the Middle East, 5 (2.4%) were Indian, 4 (1.9%) were Far-East Asian, 3 (1.4%) were US-American, 2 (1.0%) were African and 1 (0.5%) woman was originally from Western Europe. In 1 (0.5%) woman ethnicity was not disclosed. 27 (12.9%) women were smokers and none reported alcohol consumption during pregnancy. No illicit drug consumption was reported in this cohort.

**Table 1:** Baseline characteristics of women (*n* = 209) with late terminations of pregnancy due to fetal malformations at the Department of Obstetrics and Gynecology, Medical University of Vienna between 2003 and 2019

Demographics	Total 2004–2019 (*n* = 209)	Jan 2004–Dec 2011 (*n* = 57)	Jan 2012–Dec 2019 (*n* = 152)	*p* value
Maternal Age (years)^a^	30.7 ± 6.0	30.1 ± 5.9	31.0 ± 6.0	0.315^c^
University degree (*n*; %)	28 (13.4%)	6 (10.5%)	22 (14.5%)	0.456^d^
Body mass index (*n*; %)				
Underweight (≤ 18.5 kg/m^2^)	11 (6.0%)	4 (9.8%)	7 (5.0%)	0.474^d^
Normal weight (18.5–24.8 kg/m^2^)	116 (63.7%)	29 (70.7%)	87 (61.7%)	
Preobesity (25–29.9 kg/m^2^)	40 (22.0%)	7 (17.1%)	33 (23.4%)	
Obesity Class I (30–34.9 kg/m^2^)	10 (5.5%)	1 (2.4%)	9 (6.4%)	
Obesity Class II (35–39.9 kg/m^2^)	4 (2.2%)	0 (0%)	4 (2.8%)	
Obesity Class III (≥ 40 kg/m^2^)	1 (0.5%)	0 (0%)	1 (0.7%)	
Conception mode (*n*; %)				
Natural	155 (74.2%)	43 (75.5%)	112 (73.8%)	0.550^d^
ART	17 (8.1%)	4 (7.0%)	13 (8.5%)	
Missing data	37 (17.7%)	10 (17.5%)	27 (17.7%)	
Gravida^b^	2 (1–10)	2 (1–10)	2 (1–9)	0.663^c^
Para^b^	0 (0–6)	0 (0–4)	0 (0–6)	0.745^c^
Gestational age (days)^b^	176 (121–260)	176 (121–257)	176 (155–260)	0.878^c^
Fetal sex (*n*; %)				
Female	98 (46.9%)	27 (47.4%)	71 (46.7%)	0.789^d^
Male	106 (50.7%)	27 (47.4%)	79 (52.0%)	
Unknown	5 (2.4%)	3 (5.3%)	2 (1.3%)	
Fetal weight (g)^b^	753 (263–3526)	750 (440–2670)	770 (263–3526)	0.420^c^
Frequency of late terminations (*n*)	209	57	152	
Affected organ systems (*n*; %)				
Amniotic fluid	1 (0.5%)	0 (0%)	1 (0.7%)	0.650^d^
Brain/CNS	83 (39.7%)	23 (40.4%)	60 (39.5%)	
Chromosomal	33 (15.8%)	11 (19.3%)	22 (14.5%)	
Complex malformation	31 (14.8%)	7 (12.3%)	24 (15.8%)	
DiGeorge	6 (2.9%)	1 (1.8%)	5 (3.3%)	
Heart/circulatory	18 (8.6%)	5 (8.8%)	13 (8.6%)	
Infection	2 (1%)	0 (0%)	2 (1.3%)	
Metabolic	4 (1.9%)	3 (5.3%)	1 (0.7%)	
Musculoskeletal	18 (8.6%)	5 (8.8%)	13 (8.6%)	
Others	8 (3.8%)	1 (1.8%)	7 (4.6%)	
Urinary system	5 (2.4%)	1 (1.8%)	4 (2.6%)	

*ART* assisted reproductive technology

^a^Mean ± standard deviation

^b^Median (minimum–maximum)

^c^Mann–Whitney *U* test with level of significance < 0.05

^d^Fishers’ Exact test with level of significance < 0.05

Our cohort consisted of 106 (50.7%) male and 98 (46.9%) female fetuses being stillborn following feticide between 17^+3^ and 37^+1^ gestational weeks at a median age of 25^+1^ gestational weeks. In 5 (2.4%) cases, fetal sex was not disclosed.

### Prevalence and indications for late TOP over the last 16 years

**Fig. 1:**
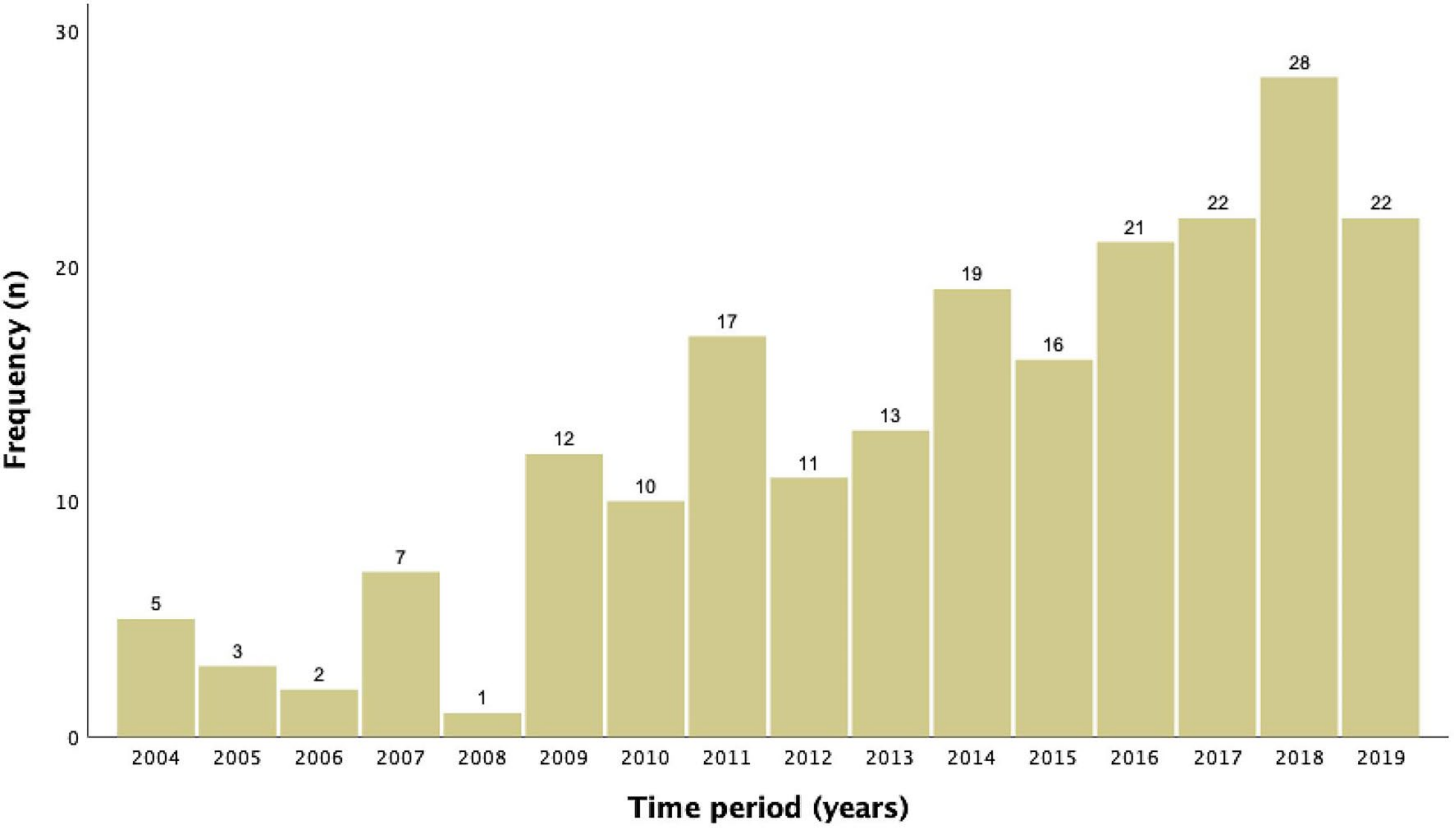
Frequency of late terminations of singleton pregnancies following feticide at the Department of Obstetrics and Gynecology, Medical University of Vienna between 2004 and 2019 (*n* = 209)

**Fig. 2:**
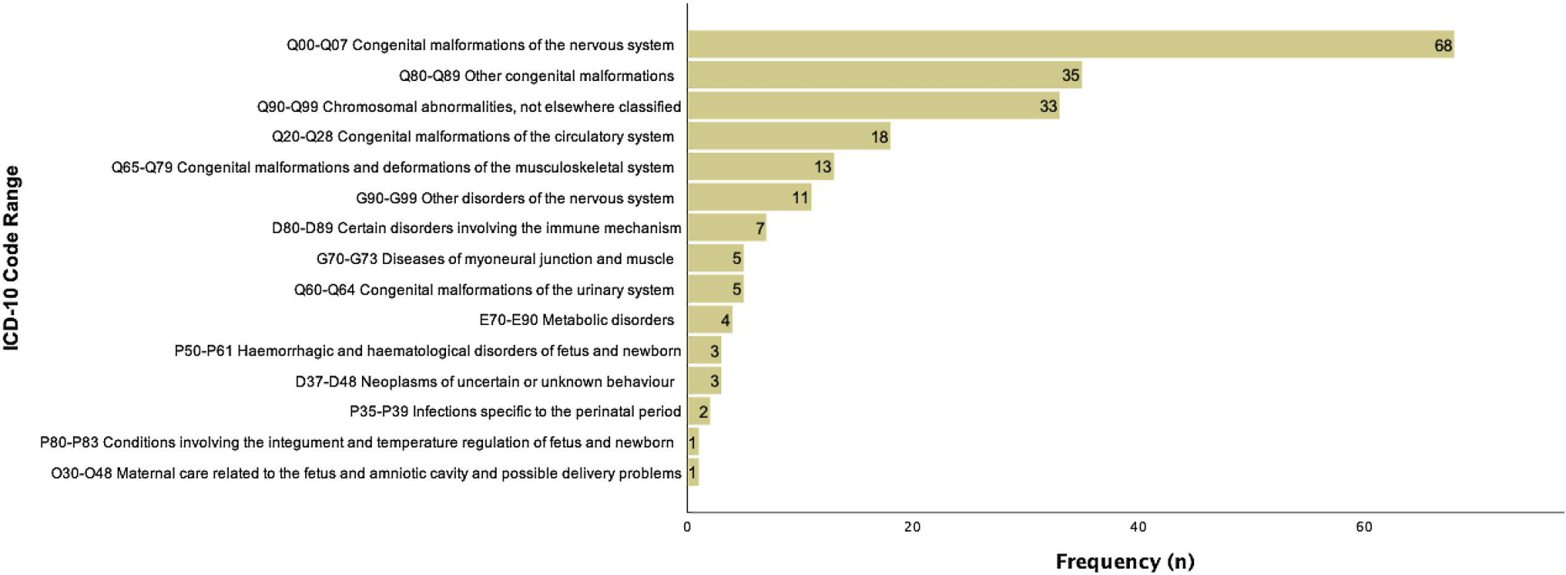
ICD-10-GM code ranges of fetal indications for late termination with feticide at the Department of Obstetrics and Gynecology, Medical University of Vienna between 2004 and 2019 (*n* = 209)

Figure 1 represents the frequency of late terminations of singleton pregnancies following feticide at our department between 2004 and 2019 (*n* = 209) and illustrates a continuous increase of late TOPs over the last 16 years, culminating in 28 late TOPs at our institution in the year 2018. The three most common fetal indications for late terminations of singleton pregnancies in the last approximately two decades were congenital malformation of the central nervous system (CNS; *n* = 68; ICD-10 GM code range Q00-Q07), “other” congenital malformations (*n* = 35; Q80-Q89) and chromosomal abnormalities (*n* = 33; Q90-Q99; Fig. 2). Table 2 shows the fetal diagnosis for feticide classified by the ICD-10 GM system in categories.

**Table 2:** ICD-10-GM categories of fetal indications for late termination by feticide at the Department of Obstetrics and Gynecology, Medical University of Vienna between 2004–2019 (*n* = 209)

ICD-10 Categories	*n*	%
Q04 Other congenital malformations of brain	45	21.5
Q89 Other congenital malformations, not elsewhere classified	24	11.5
Q90 Down syndrome	18	8.6
Q05 Spina bifida	16	7.7
G91 Hydrocephalus	11	5.3
Q24 Other congenital malformations of heart	10	4.8
Q87 Other specified congenital malformation syndromes affecting multiple systems	8	3.8
D82 Immunodeficiency associated with other major defects	7	3.3
Q03 Congenital hydrocephalus	7	3.3
Q91 Edwards syndrome and Patau syndrome	6	2.9
G71 Primary disorders of muscles	5	2.4
Q79 Congenital malformations of the musculoskeletal system, not elsewhere classified	5	2.4
Q93 Monosomies and deletions from the autosomes, not elsewhere classified	5	2.4
Q77 Osteochondrodysplasia with defects of growth of tubular bones and spine	4	1.9
E84 Cystic fibrosis	3	1.4
Q85 Phakomatoses, not elsewhere classified	3	1.4
Q92 Other trisomies and partial trisomies of the autosomes, not elsewhere classified	3	1.4
D48 Neoplasm of uncertain or unknown behaviour of other and unspecified sites	2	1.0
Q20 Congenital malformations of cardiac chambers and connections	2	1.0
Q21 Congenital malformations of cardiac septa	2	1.0
Q60 Renal agenesis and other reduction defects of kidney	2	1.0
Q64 Other congenital malformations of urinary system	2	1.0
Q72 Reduction defects of lower limb	2	1.0
P52.9 Intracranial (nontraumatic) haemorrhage of fetus and newborn, unspecified	1	0.5
D43 Neoplasm of uncertain or unknown behaviour of brain and central nervous system	1	0.5
E83 Disorders of mineral metabolism	1	0.5
O41 Other disorders of amniotic fluid and membranes	1	0.5
P35 Congenital viral diseases	1	0.5
P37 Other congenital infectious and parasitic diseases	1	0.5
P52.4 Intracerebral (nontraumatic) haemorrhage of fetus and newborn	1	0.5
P61 Other perinatal haematological disorders	1	0.5
P83 Other conditions of integument specific to fetus and newborn	1	0.5
Q22 Congenital malformations of pulmonary and tricuspid valves	1	0.5
Q23 Congenital malformations of aortic and mitral valves	1	0.5
Q25 Congenital malformations of great arteries	1	0.5
Q28 Other congenital malformations of circulatory system	1	0.5
Q61 Cystic kidney disease	1	0.5
Q73 Reduction defects of unspecified limb	1	0.5
Q78 Other osteochondrodysplasias	1	0.5
Q99 Other chromosome abnormalities, not elsewhere classified	1	0.5

In total, severe congenital malformations affected the brain/CNS in 83 (39.7%) cases, heart/circulatory system in 18 (8.6%) cases, musculoskeletal system (including diaphragm) in 18 (8.6%) cases, genito-urinary system in 5 (2.4%) cases, chromosomal abnormalities in 33 (15.8%) cases, genetically confirmed DiGeorge syndrome in 6 (2.9%) cases, metabolic conditions in 4 (1.9%) cases, congenital infection in 2 (1.0%) cases, detrimental amniotic fluid changes in 1 (0.5%) case, “others” in 8 (3.8%) cases and complex multi-organ malformations in 31 (14.8%) cases.

### Temporal changes in feto-maternal parameters and indication

Between 1 January 2004 and 31 December 2011, 57 singleton pregnancies were terminated late, whereas between 1 January 2012 and 31 December 2019, the cumulative incidence had increased by 2.6-fold to 152 late TOPs with feticide at our institution. Comparing the two time intervals, we found no significant differences regarding maternal age (30 ± 6 years vs 31 ± 6 years; *p* = 0.315), methods of conception (*p* = 0.550), median gestational age at termination [176 (121–257) days vs. 176 (155–260) days; *p* = 0.878] nor median fetal birthweight [750 (440–2670) g vs. 770 (263–3526) g; *p* = 0.420].

Regarding the spectrum of fetal indications, there was no significant difference neither in ICD-10-GM codes [*p* = 0.915; 99% CI (0.908–0.922)] nor in affected organ systems [*p* = 0.650; 99% CI (0.639–0.662)] over time.

**Fig. 3:**
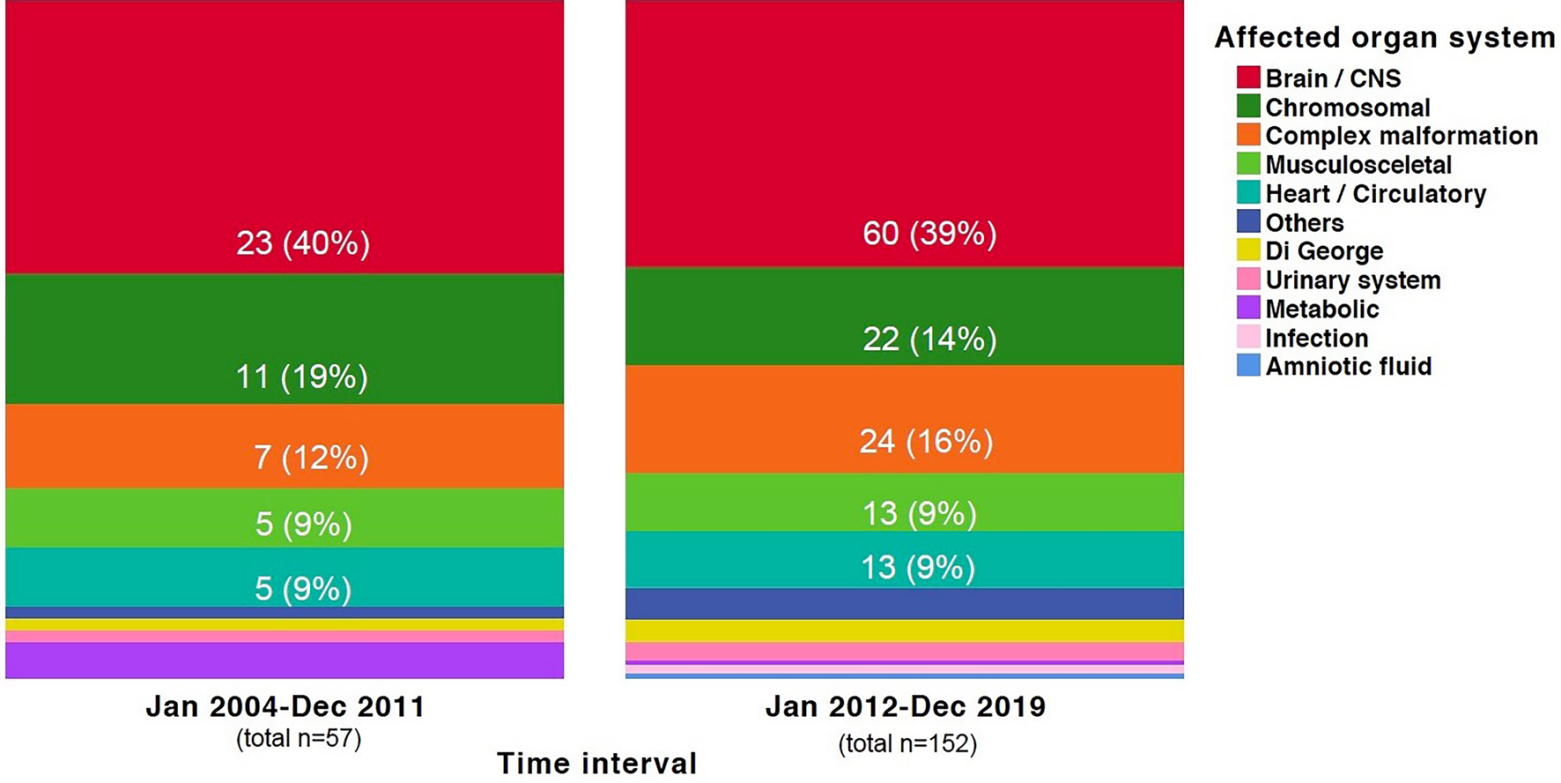
Frequency of fetal organ systems (number; percentage) affected by congenital malformations leading to late terminations of singleton pregnancy between 2004 and 2019 (*n* = 209)

The four main affected organ systems for late TOP with feticide between 2004 and 2011 as well as 2012 and 2019 were brain/CNS (*n* = 23; 40.4% vs. *n* = 60; 39.5%), chromosomal aberrations (*n* = 11; 19.3% vs. *n* = 22; 14.5%), complex malformations (*n* = 7; 12.3% vs. *n* = 24; 15.8%) and abnormaltities of the musculoskeletal system including diaphragmatic hernias (*n* = 5; 8.8% vs. *n* = 13; 8.6%); Fig. 3].

## Discussion

By this study, we show that the overall performance of prenatal diagnosis, detection of malformations and, thus, late terminations have continuously increased in frequency over time. After all, maternal age remained normally distributed throughout the study period and in each interval, reflecting the average maternal age of approximately 30 years at first childbirth in Central Europe. Likewise, the prevalence of higher education as well as the median BMI has not changed significantly over time in our cohort. Regarding fetal parameters including indications for TOP and gestational age at termination, no tendency was observed within two decades. The same applies with the stable incidences of chromosomal aberrations, such as trisomy 21, 13 or 18, as indications for late TOP. Despite the introduction of cell-free DNA testing at our institution about 10 years ago and its regular use ever since, especially in conjunction with first-trimester biochemical and ultrasound screening, the incidence of late terminations for aneuploidy remained constant.

Previous studies have accounted the improved technical and personal skills in ultrasound screening for the continuously raising detection rates of fetal abnormalities over the recent years at individual institutions [16–18]. The introduction of prenatal ultrasound screening in the mid 1980s and the continuous quality audits by respective supervising organs in perinatal medicine, allow us to negate any effect with regard to improved detection rates.

Considering the spectrum of fetal congenital malformations in late TOPs, our results support previous retrospective studies which confirmed brain malformations being the leading cause for late TOPs in many parts of the world [18–22]. One important reason for this is the late cortical formation and maturation and therefore delayed and progressive development of brain malformations up until the late second and third trimesters [23]. Several studies have already addressed the difficulty of predicting neonatal outcome following live birth of a child with congenital brain anomalies [1, 24]. It, therefore, remains a big emotional dilemma and ethical-moral debate as to whether natural course of disease should be allowed postnatally, taking natural death into account, or whether to induce medical late abortion. Taking these data together, fetal medicine may remain confronted with belated diagnoses of CNS anomalies also in the future. This should prompt practice-changing developments in fetal MRI together with artificial intelligence for earlier detection and accurate prediction of neonatal outcome following brain defects.

The strengths of our study are its strict inclusion and exclusion criteria, resulting in a homogenous cohort limited to late terminations of singleton pregnancy by medical assistance whilst omitting selective fetal reductions in higher-grade pregnancies. Furthermore, the inclusion of a single center, in which the same individuals have constituted the ethical board for the last two decades, allows to neglect any alteration in group-collective ethical judgments in favor for or opposed to fetal indications for late TOPs with feticide over time.

Last but not least, the thorough review of all prenatal reports (including ultrasound and magnetic resonance scans, genetic tests) and post-mortem autopsy reports allowed us to define the ICD-10 code for each individual case with high accuracy and validation.

Despite these strengths, we acknowledge certain study limitations. Inherent to its single-center setting, the total number of our cohort was relatively small with an unequal number in the two time periods. Furthermore, due to its retrospective study design, we were unable to control for maternal demographic parameters, as these were self-reported by the woman and therefore subject to recall bias at time of antenatal booking.

Our results draw the current profile of maternal–fetal characteristics in Austria. The justification of our study increasingly expands to the unforeseen demographic changes within Central Europe. Due to the increasing pressure of local restrictions in abortion laws, it is expected, that “abortion-tourism” will raise in the coming years, introducing a shift in maternal and fetal parameters. It would be, therefore, be, socio-medically valid to compare our present data (as of December 2019) with new data that will be generated within the next years to better understand demographic patterns within the European context and sketch the changing epidemiological landscape of late TOPs.

In conclusion, our study shows that despite a temporal increase in late terminations of singleton pregnancies, maternal and fetal profiles have broadly remained constant within the last two decades.

## Declarations

### Availability of data and material

Available upon request.
